# Supplementary material for: Mechanics and dynamics of translocating MreB filaments on curved membranes
Source: eLife. 2019 Feb 18;8:e40472. doi: 10.7554/eLife.40472 (PMC6504236; doi:10.7554/eLife.40472)
Supplement: Supplementary file 1. [file elife-40472-supp1.pdf]

Quantity	Estimate	Source
**MreB values**		
MreB filament length, L_f	220 nm	[1]
	100-500 nm	[1–6]
MreB monomer length, ℓ_f	51 Å	[3,7]
MreB cross-sectional radius, r_f	3.2 nm	[3]
MreB filament intrinsic radius of curvature, R_s	300 nm	[1]
	200-300 nm	[1–3]
MreB Young's modulus, Y	similar to actin; 2 GPa	[8]
MreB cross-sectional binding fraction, b	1/6	this work
	0	[1]
MreB filament flexural rigidity, B	$\frac{\pi Y r_f^4}{4} \approx 1.65 \times 10^{-25}$ J $\cdot$ m	–
**Membrane values**		
Lipid bilayer bending modulus, k_b	10 kT	[9]
	10-20 kT	[9]
Pressure difference across membrane in *E. coli* (*B. subtilis*), p	1 atm (20 atm)	[10,11]
	0.3-2 atm	[10,12,13]
Typical cell radius for *E. coli* and *B. subtilis*, R_{cell}	500 nm	[1,14]
	400-600 nm	[1,6,14,15]
**Binding energy values**		
Unit MreB monomer-membrane interaction energy, ε_0	10 kT	[1]
Number of membrane binding sites, N_{int}	$2 \times L_f/\ell_f \approx 86$	–
Temperature, T	300 K	–

Supplementary file 1: Variables used, or calculated, in the model of filament binding and their numerical values. The first value listed is the value assumed in this work, and subsequent values, when available, indicate estimated ranges for the same variable. Note that refs. [2,3,7] refer to *Thermotoga maritima* MreB and ref. [5] refers to *Caulobacter crescentus* MreB.

[1] Hussain, S. *et al.* MreB filaments align along greatest principal membrane curvature to orient cell wall synthesis. *eLife* **7**, e32471 (2018).

[2] Salje, J., van den Ent, F., de Boer, P. & Löwe, J. Direct membrane binding by bacterial actin MreB. *Mol. Cell* **43**, 478–487 (2011).

[3] van den Ent, F., Izoré, T., Bharat, T. A. M., Johnson, C. M. & Löwe, J. Bacterial actin MreB forms antiparallel double filaments. *eLife* **3**, e02634 (2014).

[4] Olshausen, P. V. *et al.* Superresolution imaging of dynamic MreB filaments in *B. subtilis*—a multiple-motor-driven transport? *Biophys. J.* **105**, 1171–1181 (2013).

[5] Kim, S. Y., Gitai, Z., Kinkhabwala, A., Shapiro, L. & Moerner, W. E. Single molecules of the bacterial actin MreB undergo directed treadmilling motion in *Caulobacter crescentus*. *Proc. Nat. Acad. Sci. USA* **103**, 10929–10934 (2006).

[6] Ouzounov, N. *et al.* MreB orientation correlates with cell diameter in *Escherichia coli*. *Biophys. J.* **111**, 1035–1043 (2016).

[7] van den Ent, F., Amos, L. A. & Löwe, J. Prokaryotic origin of the actin cytoskeleton. *Nature* **413**, 39–44 (2001).

[8] Kojima, H., Ishijima, A. & Yanagida, T. Direct measurement of stiffness of single actin filaments with and without tropomyosin by *in vitro* nanomanipulation. *Proc. Natl. Acad. Sci. USA* **91**, 12962–12966 (1994).

[9] Phillips, R., Kondev, J., Theriot, J. & Garcia, H. *Physical Biology of the Cell* (Garland Science, 2012).

[10] Cayley, D. S., Guttman, H. J. & Record, M. T. Biophysical characterization of changes in amounts and activity of *Escherichia coli* cell and compartment water and turgor pressure in response to osmotic stress. *Biophys. J.* **78**, 1748–1764 (2000).

[11] Whatmore, A. M. & Reed, R. H. Determination of turgor pressure in *Bacillus subtilis*: a possible role for K+ in turgor regulation. *J. Gen. Microbiol.* **136**, 2521–2526 (1990).

[12] Deng, Y., Sun, M. & Shaevitz, J. W. Direct measurement of cell wall stress stiffening and turgor pressure in live bacterial cells. *Phys. Rev. Lett.* **107**, 158101 (2011).

[13] Koch, A. L. The surface stress theory for the case of *Escherichia coli*: the paradoxes of Gram-negative growth. *Res. Microbiol.* **141**, 119–130 (1990).

[14] Wong, F. *et al.* Mechanical strain sensing implicated in cell shape recovery in *Escherichia coli*. *Nat. Microbiol.* **2**, 17115 (2017).

[15] Furchtgott, L., Wingreen, N. S. & Huang, K. C. Mechanisms for maintaining cell shape in rod-shaped Gram-negative bacteria. *Mol. Microbiol.* **81**, 340–353 (2011).
