# Supplementary material for: Mechanics and dynamics of translocating MreB filaments on curved membranes
Source: eLife. 2019 Feb 18;8:e40472. doi: 10.7554/eLife.40472 (PMC6504236; doi:10.7554/eLife.40472)
Supplement: Supplementary file 2. [file elife-40472-supp2.pdf]

Quantity	Dimensional Estimate	Simulation Value	Source
MreB translocation step size, L	200 nm	0.4	this work
	30 nm-1 μm	–	this work
Time per step	$L/v = 20$ s (7 s)	–	–
Cell radius, a	0.5 μm	1	–
Typical cell length	2 μm	4	–
Number of steps, N	~ 20 min	60 (170)	–
Filament speed, v	10 nm/s (30 nm/s)	–	[1–3]
	5-30 nm/s (20-85 nm/s)	–	[1–7]
Time per step	$L/v = 20$ s (7 s)	–	–
Activation rate, k	$\sim 20\ \mu\text{m}^{-2} \cdot\ \text{min}^{-1}$	40 (15)	[8]
MreB filament persistence time, τ	5 min	–	[9]
Deactivation rate, λ	$1/\tau = 0.2\ \text{min}^{-1}$	0.07 (0.02)	–
Linear dependence of σ on Δc, α	(0.6 rad $\cdot\ \mu\text{m}^{-1}$)	0.3	–
Quadratic dependence of σ on Δc, β	(0.6 rad $\cdot\ \mu\text{m}^{-2}$)	0.15	–
Typical translocation noise, σ	0.3 rad	0.3	[1]
	0.1-0.5 rad	–	[1,3,10]

Supplementary file 2: Variables used, or calculated, in the model of filament translocation and their numerical values for *E. coli*. Values exclusive to *B. subtilis*, when relevant, are in parentheses, and dashes denote values that are not used or not relevant. The first value listed is the value assumed in this work, and subsequent values, when available, indicate estimated ranges for the same variable.

[1] Hussain, S. *et al.* MreB filaments align along greatest principal membrane curvature to orient cell wall synthesis. *eLife* **7**, e32471 (2018).

[2] van Teeffelen, S. *et al.* The bacterial actin MreB rotates, and rotation depends on cell-wall assembly. *Proc. Natl. Acad. Sci. USA* **108**, 15822–15827 (2011).

[3] Garner, E. C. *et al.* Coupled, circumferential motions of the cell wall synthesis machinery and MreB filaments in *B. subtilis*. *Science* **333**, 222–225 (2011).

[4] Olshausen, P. V. *et al.* Superresolution imaging of dynamic MreB filaments in *B. subtilis*—a multiple-motor-driven transport? *Biophys. J.* **105**, 1171–1181 (2013).

[5] Domínguez-Escobar, J. *et al.* Processive movement of MreB-associated cell wall biosynthetic complexes in bacteria. *Science* **333**, 225–228 (2011).

[6] Reimold, C., Soufo, H. J. D., Dempwolff, F. & Graumann, P. L. Motion of variable-length MreB filaments at the bacterial cell membrane influences cell morphology. *Mol. Biol. Cell* **24**, 2340–2349 (2013).

[7] Billaudeau, C. *et al.* Contrasting mechanisms of growth in two model rod-shaped bacteria. *Nat. Commun.* **8**, 15370 (2017).

[8] Wong, F. *et al.* Mechanical strain sensing implicated in cell shape recovery in *Escherichia coli*. *Nat. Microbiol.* **2**, 17115 (2017).

[9] Ursell, T. S. *et al.* Rod-like bacterial shape is maintained by feedback between cell curvature and cytoskeletal localization. *Proc. Natl. Acad. Sci. USA* **111**, 1025–1034 (2014).

[10] Ouzounov, N. *et al.* MreB orientation correlates with cell diameter in *Escherichia coli*. *Biophys. J.* **111**, 1035–1043 (2016).
